# Influence of nebivolol on anticonvulsant effect of lamotrigine

**DOI:** 10.4103/0253-7613.48890

**Published:** 2009-02

**Authors:** Radha Goel, Amit Goel, Anshu manocha, K.K. Pillai, Rashmi S. Srivastava

**Affiliations:** ITS Paramedical College, Ghaziabad, India; 1 Jamia Hamdard, New Delhi-110 062, India

**Keywords:** Antiepileptic drugs, grip-strength test, increasing current electroshock seizures, SAB

## Abstract

**Objective:**

The present study describes the effect of nebivolol (NBV) either alone or in combination with lamotrigine (LTG) using increasing current electroshock seizures (ICES) model in mice.

**Materials and Methods:**

Male albino mice of Swiss strain each weighing 18-30 g were used. Lamotrigine (Lamitor tablets, Torrent; 1.5 and 3.0 mg/kg) and NBV (Nebicard tablets, Torrent; 0.25 and 0.5 mg/kg) were suspended in 0.25% of carboxy methyl cellulose (CMC) in 0.9% saline and administered orally in volumes of 10 mg/kg. Control animals received an equivalent volume of 0.25% CMC in 0.9% saline suspension. The anticonvulsant effects of the drugs were measured using ICES model whereas cognitive behavior was measured by the spontaneous alternation behavior and grip-strength test. The biochemical estimation was done by measuring the lipid peroxidation and reduced glutathione (GSH).

**Results:**

Both NBV and LTG produced significantly enhanced seizure threshold (ST), decreased grip strength, inhibited lipid peroxidation, and increased brain GSH levels in acute and chronic dosages likened to control group, whereas there was no significant effect on alternation scores. The combination of NBV with LTG significantly potentiated the ST when compared to LTG.

**Conclusion:**

Nebivolol showed antiepileptic effects in addition to its reported antihypertensive effect, which could be attributed to action of the two drugs through different mechanisms or due to drug interaction that may be pharmacodynamic or pharmacokinetic needing elucidation.

## Introduction

Epilepsy is the most common serious neurological disorder affecting an estimated 50 million people worldwide. So, for appropriate antiepileptic drugs (AEDs), the identification of the epilepsy syndrome is important.[1] Hypertension can lead to seizures through vascular brain damage that might or might not involve manifest stroke.[2] This relationship suggests that hypertension, particularly severe and uncontrolled, might increase the risk of epilepsy in the absence of prior clinically detected stroke;[3] postulate that hypertension raises the risk of new-onset seizures in the absence of an immediate stroke.[2] The striking synergism between hypertension and stroke is more epileptogenic than other risk factors.[4] The contribution of noradrenergic transmission to the seizure susceptibility and epileptogensis is gaining more attention lately. The involvement of noradrenergic system in modulation of seizure activity is well documented. The noradrenergic system was demonstrated to participate in the occurrence of seizure in epileptic EL mice and to increase epileptiform discharge in rat limbic system via β-adrenergic receptor stimulation.[5]

Lamotrigine (LTG), a new antiepileptic drug blocks voltage-dependent sodium channel and reduces excitatory neurotransmitter release, principally that of glutamate leaving normal glutamate release unaffected. Its ED50 is 6 mg/kg.

Nebivolol (NBV) is a β-1-adrenoceptor-blocking agent.[6] NBV is a racemic mixture of equal amounts of D-NBV and L-NBV. The D-isomer is a potent, highly selective and long acting β-1-adrenoceptor-blocking agent.[7] NBV has antioxidative effect[8] and is a highly lipophilic drug.[9]

Epilepsy is frequently associated with impaired memory and muscle relaxation. Such patients would therefore need additional treatment besides AED therapy, to correct the accompanying neurological deficits.

The aim of the study was to find an effective and efficient drug combination for cases where risk factors like hypertension and/or stroke might precipitate seizures, especially for the elderly who are at risk of cerebrovascular-induced seizures, leading to seizure freedom and restoration of confidence and independence.

## Materials and Methods

### Animals

Male albino mice, of Swiss strain each weighing 18-30 g, were used. Animals were housed in groups of 5-10 mice per cage, maintained at 20-30 °C, 50-55% humidity in natural light-dark cycle, with free access to food and water. The experiments were performed during the light cycle in awake, freely moving animals that were acclimatized to laboratory conditions preceding the experiments. Animals were procured from the central animal house, approved by the animal ethics committee Jamia Hamdard, New Delhi.

### Drugs

The following drugs were used in the study: LTG (Lamitor tablets, Torrent), and NBV (Nebicard tablets, Torrent). Drugs were suspended in 0.25% carboxy methyl cellulose (CMC) in 0.9% saline suspension, and were freshly prepared prior to administration. All drugs were given in volumes of 10 ml/kg p.o. The doses of LTG were selected half and one-fourth of the ED50 (1.5 and 3 mg/Kg), while the doses of NBV were selected one-tenth and one-twentieth of human dose[10] (0.25 and 0.5 mg/Kg). Control group received an equivalent volume of 0.25% CMC in 0.9% saline suspension.[11] To study the chronic effects, the drugs were administered for 21 days. All observations were made 1.5 hours after LTG treatment (on the same day for studying acute effects and on the twenty first day for chronic effects) and 0.5 hours after NBV treatment, to correspond with the peak plasma concentrations for either drug. Observations from control groups were pooled together for a combined control group for each test (increasing current electroshock seizures (ICES), SAB, grip-strength test (GST), TBARS, and GSH. There were six mice per group. Each mouse underwent only one test and was not reused.

### Statistical analysis

The results were presented as mean ± standard error of mean (SEM). ANOVA and Dunnetts ‘*t* test using graph pad instat were used for data analysis. ‘*P*’ values < 0.05 were considered significant.

## Experimental procedures

### Increasing current electroshock seizures[12]

The ICES test was used to evaluate the anticonvulsant effect of the drugs. Starting with a current of 2 mA, electric shock was delivered to each animal via ear electrodes as a single train of pulses (for 0.2 sec) with linearly increasing intensity of 2 mA/2 sec. The current at which tonic hindlimb extension occurred was recorded as ST current. If no tonic hindlimb extension was observed up to a current of 30 mA, electric shock was terminated and this cut-off current was used in the analysis.

### Spontaneous alternation behavior on plus maze[13]

Cognitive function was assessed by measuring percentage alternation on a plus maze consisting of four arms (height: 50 cm; length: 23.5 cm, breadth: 8 cm; and wall height: 10 cm) with a central platform (8 × 8 cm). The arms were labeled as A, B, C, and D, and after being placed in the central platform, mice were allowed to move freely in the maze for six minutes. The number and sequence of entries were recorded. A 4/5 alternation was defined as entry into four different arms on overlapping quintuple sets. Five consecutive arm choices made up a quintuple set, for example, a quintuple set consisting of arm choices A, B, C, D, and B was considered as an alternation, while A, D, C, D, and A were not considered as quintuple. Using these procedures percentage alternation was calculated as the ratio of actual number of alternations with possible number of alternations (number of arm entries – 4):

Percentage alternation = (Actual number of alternations/Possible alternations) × 100

where possible alternation = number of arm entries – 4

## Grip-strength test[14]

A grip strength meter was used to measure the forelimb grip strength, as an indicator of muscular function. The grip strength meter was positioned horizontally and mice were held by the tail and lowered toward the apparatus. Mice were allowed to grasp the smooth metal rectangular pull bar (with forelimbs only) and were then pulled backwards in the horizontal plane. The force applied to the bar at the moment grasp was released was recorded as the peak tension (kg). The test was repeated for five consecutive times within the same session and the highest value from the five trials was recorded as the grip strength for that animal. Mice were not trained prior to testing and each animal was tested only once.

### Lipid peroxidation in brain[15]

One milliliter of suspension medium was taken from 10% of tissue homogenate. To this, 1 ml of 30% TCA was added, followed by 1 ml of 0.8% TBA reagent. The tubes were covered with aluminum foil and kept in a shaking water bath for 30 minutes at 80 °C. After 30 minutes, the tubes were taken out and kept in ice-cold water for 30 minutes. These were then centrifuged at 3000 rpm for 15 minutes. The absorbance of the supernatant was read at 535 nm at room temperature against appropriate blank. Blank consisted of 1 ml distilled water, 1ml of 30% TCA, and 1ml of 0.8%TBA.

The content of MDA, expressed as *n* moles formed per milligram of protein in the tissue, was calculated using the formula:

Concentration = A*(*V*/E)*P

Where *A* is absorbance, *V* is the volume of the solution, E is extinction coefficient (1.56*110^−6^ m^−1^ cm^−1^), and *P* is the protein content of tissue calculated as milligram of protein per gram of tissue.

### Brain glutathione[16]

To 2 ml of 10% homogenate, which was prepared in KCl solution, 2.5 ml of 0.02 M EDTA was added and shaken vigorously. To 2 ml of this mixture 4 ml of cold distilled water and 1 ml of 50% TCA were added and shaken for 10 minutes. Thereafter, the contents were centrifuged at 3000 rpm for 15 minutes. Following centrifugation, 2 ml of the supernatant was mixed with 4 ml of 0.4 M tris buffer (Ph 8.9). The whole solution was mixed well and 0.1 ml of 0.01 M DTNB was added, the absorbance was read within 5 minutes of addition of DTNB at 412 nm against reagent blank with no homogenate. For blank readings, the homogenate was substituted by 2 ml of distilled water.

Total glutathione (GSH) (tissue) was calculated using the formula:

Co = (A*D)/E

Where *A* is absorbance at 412 nm, *D* is dilution factor, and E is the molar extinction coefficient (C = 13000M-1cm-1); Co is the concentration of GSH.

## Results

### Effects of lamotrigine and nebivolol in increasing current electroshock seizures

#### Acute effects

On acute administration, NBV (0.25 mg/kg) did not significantly enhance seizure threshold (ST) as compared to control group, whereas LTG (1.5 and 3.0 mg/kg) and NBV (0.5 mg/kg) alone significantly enhanced the ST in the ICES test [Table 1]).

**Table 1:** Effect of acute and chronic administration of lamotrigine and nebivolol on seizure threshold in the increasing current electroshock seizures test in mice

*Group*	*Drug*	*Dose (mg/kg; p.o.)*	*Seizure threshold Current (mA ± SEM) Acute dosage*	*Seizure threshold Current (mA ± SEM) Chronic dosage*
I	Control (CMC in Saline)	10 ml/kg/p.o.	12.8 ± 0.40	14.66 ± 0.33
II	LTG	1.5	20 ± 0.33*	24.33 ± 0.33*
III	LTG	3.0	23.6 ± 0.802*	26.66 ± 1.11*
IV	NBV	0.25	15 ± 1.0	21.66 ± 0.808*
V	NBV	0.5	20.6 ± 0.98*	23.33 ± 2.171*
VI	LTG + NBV	1.5 + 0.25	25.3 ± 2.11**	26.33 ± 2.23**
VII	LTG + NBV	1.5 + 0.5	27.3 ± 2.68**	28.66 ± 0.988**
	*F* value		12.478	11.157

Data expressed as mean ± SEM, p.o.: per os;

* *P* < 0.01 vs, group I (control)

** *P* < 0.01 vs. group II (LTG: 1.5 mg/kg)

The combination of LTG (1.5 mg/kg) plus NBV (0.25 mg/kg and 0.5 mg/kg) resulted in significant enhancement of ST as compared to LTG (1.5 mg/kg) alone.

#### Chronic effects

Of the chronic effect, both the doses of LTG and NBV significantly enhanced the ST when compared to control.

The combination of LTG (1.5 mg/kg) plus NBV (0.25 mg/kg and 0.5 mg/kg) resulted in significant enhancement of ST as compared to LTG (1.5 mg/kg) alone.

### Effects of lamotrigine and nebivolol in grip-strength test

Of acute as well as chronic effects of the drugs in the study, LTG and NBV alone in both the doses showed significant decrease in grip strength likened to control (*P*<0.01), and the combination of LTG (1.5 mg/kg) with both the doses of NBV (0.25 and 0.5 mg/kg) showed significant decrease in the grip strength test as compared to LTG (1.5 mg/kg) (*P*<0.01) and control. There was no significant difference in grip strength in the groups, which received lower and higher doses of LTG and NBV [Table 2].

**Table 2:** Effect of acute and chronic administration of lamotrigine and nebivolol on grip-strength test in mice

*Group*	*Drug*	*Dose (mg/kg; p.o.)*	*Force Kg ± SEM Acute study*	*Force Kg ± SEM Chronic study*
I	Control (CMC in saline)	10 ml/kg/p.o.	0.143 ± 0.006	0.095 ± 0.002
II	LTG	1.5	0.118 ± 0.002*	0.062 ± 0.002*
III	LTG	3.0	0.097 ± 0.005*	0.057 ± 0.005*
IV	NBV	0.25	0.105 ± 0.006*	0.054 ± 0.004*
V	NBV	0.5	0.115 ± 0.008*	0.060 ± 0.005*
VI	LTG + NBV	1.5 + 0.25	0.089 ± 0.002**	0.040 ± 0.008**
VII	LTG + NBV	1.5 + 0.5	0.080 ± 0.004**	0.033 ± 0.008**
	*F* value		7.235	5.799

Data expressed as mean ± SEM, p.o.: per os

* *P* < 0.01 vs. group I (control)

** *P* < 0.01 vs. group II (LTG: 1.5 mg/kg)

### Effects of lamotrigine and nebivolol on spontaneous alternation behavior

#### Acute effects

Acute administration of LTG (1.5 and 3 mg/kg) and NBV (0.25 and 0.5 mg/kg) showed moderate decrease in the alternation score, but was insignificant (*P* > 0.05).

The combination of LTG (1.5 mg/kg) with both the doses of NBV (0.25 and 0.5 mg/kg) did not show significant difference as compared to LTG (1.5 mg/kg) alone and control [Table 3].

**Table 3:** Effect of acute and chronic administration of lamotrigine and nebivolol on spontaneous alternation behavior in mice

*Group*	*Drug*	*Dose (mg/kg; p.o.)*	*Percentage alternation ± SEM Acute effect*	*Percentage alternation ± SEM Chronic effect*
I	Control (CMC in saline)	10 ml/kg/p.o.	67.08 ± 4.86	62.1 ± 9.439
II	LTG	1.5	65.7 ± 13.74	74 ± 9.801
III	LTG	3.0	63.6 ± 11.01	78.23 ± 5.41
IV	NBV	0.25	58.06 ± 8.18	71.43 ± 5.75
V	NBV	0.5	66.01 ± 5.97	73.56 ± 10.60
VI	LTG + NBV	1.5 +0.25	69.48 ± 3.620	62.69 ± 7.43
VII	LTG + NBV	1.5 +0.5	68.62 ± 4.665	47.38 ± 8.82

Data expressed as mean ± SEM, p.o.: per os

* *P* < 0.05 vs. group I (control)

** *P* < 0.05 group II (LTG: 1.5 mg/kg)

#### Chronic effects

Chronic administration of LTG (1.5 and 3.0 mg/kg) and NBV (0.25 and 0.5 mg/kg) moderately increased the alternation score, but was not significant (*P* > 0.05).

The combination of the LTG (1.5 mg/kg) with both the doses of NBV (0.25 and 0.5 mg/kg) also showed no significant decrease in alternation scores when compared to LTG (1.5 mg/kg) and control (*P* > 0.05) [Table 3].

### Lipid peroxidation in brain

#### Acute and chronic effects

On acute as well as in chronic administration of LTG (1.5 and 3.0 mg/kg) and NBV (0.25 and 0.5 mg/kg) alone showed significant inhibition of lipid peroxidation compared to control (*P* < 0.01) as well as in combination of LTG with both the doses of NBV (0.25 and 0.5 mg/kg) showed significant inhibition of lipid peroxidation likened to LTG (1.5 mg/kg) and control (*P* < 0.01) [Table 4].

**Table 4:** Effect of acute and chronic administration of lamotrigine and nebivolol on lipid peroxidation in mice brain tissue

Group	Drug	Dose (mg/kg; p.o.)	*n* moles of MDA/mg of protein *Acute effect*	*n moles of MDA/mg of protein Chronic effect*
I	Control (CMC in Saline)	10 ml/kg/p.o	0.466 ± 0.030	1.25 ± 0.129
II	LTG	1.5	0.326 ± 0.0166*	0.921 ± 0.032*
III	LTG	3.0	0.290 ± 0.007*	0.765 ± 0.050 *
IV	NBV	0.25	0.276 ± 0.028*	0.531 ± 0.022*
V	NBV	0.5	0.258 ± 0.020*	0.503 ± 0.035*
VI	LTG + NBV	1.5 + 0.25	0.210 ± 0.006**	0.45 ± 0.048**
VII	LTG + NBV	1.5 + 0.5	0.199 ± 0.018**	0.43 ± 0.054**
	*F* value		15.758	23.142

Data expressed as mean ± SEM, p.o.: per os

* *P* < 0.01 vs. group I (control)

** *P* < 0.01 vs. group II (LTG: 1.5 mg/kg)

### Reduced glutathione in brain

#### Acute effect

On acute administration, LTG (1.5 mg/kg) and NBV (0.25 and 0.5 mg/kg) showed no significant increase in brain GSH level compared to control (*P* > 0.01), but LTG (3.0 mg/kg) showed significant increase in brain GSH level, whereas the combination of both the drugs, that is, LTG with both the doses of NBV showed significant effect when compared to LTG (1.5 mg/kg) alone and control *(P* < 0.01) [Table 5].

**Table 5:** Effect of acute and chronic administration of lamotrigine and nebivolol on glutathione level in mice brain tissue

*Group*	*Drug*	*Dose (mg/kg; p.o.)*	*Micro mole/mg of protein Acute effect*	*Micro mole/mg of protein Chronic effect*
I	Control (CMC in saline)	10 ml/kg/p.o.	18.33 ± 1.382	21.33 ± 3.630
II	LTG	1.5	27.83 ± 4.159	39.5 ± 3.085*
III	LTG	3.0	42 ± 1.291*	43.83 ± 2.344*
IV	NBV	0.25	26.16 ± 7.616	49.66 ± 3.232*
V	NBV	0.5	26 ± 7.929	46.66 ± 4.137*
VI	LTG + NBV	1.5 + 0.25	49.5 ± 3.964**	59.5 ± 5.353**
VII	LTG + NBV	1.5 + 0.5	52.33 ± 3.818**	60.33 ± 3.029**
	*F* value		5.698	10.710

Data expressed as mean ± SEM, p.o.: per os

* *P* < 0.01 vs. group I (control)

** *P* < 0.01 vs. group II (LTG: 1.5 mg/kg)

#### Chronic effect

On chronic administration, LTG (1.5 and 3.0 mg/kg) and NBV (0.25 and 0.5 mg/kg) alone showed significant increase in the level of brain GSH when compared to (*P* < 0.01) as well as in combination of lower dose of LTG with both the doses of NBV showed significant increase in the level of brain GSH when compared to LTG (1.5 mg/kg) and control (*P* < 0.01).

## Discussion

The present study confirms the antiepileptic efficacy of LTG as already reported.[11,15] Additionally, it opens some interesting neurobehavioral possibilities with LTG, given alone or in combination with NBV.

In the present study, acute treatment with NBV at a lower dose (0.25 mg/kg) did not affect the ST, but a higher dose (0.5 mg/kg) exhibited significant enhancement. On chronic administration, NBV (0.25 and 0.5 mg/kg) demonstrated the anticonvulsant effect in ICES model. NBV is a selective β-1-adrenoceptor blocker. Thus, there is a possibility that the anticonvulsant effect of NBV may be due to blockade of β-adrenoceptor. Moreover, β-receptor-mediated increase in cAMP levels potentiates glutamatergic transmission.[17] β-adrenergic blockade leads to the reduced formation of cAMP; it may be hypothesized that β-adrenoceptor antagonist potentiates the activity of AED that do not diminish the cAMP levels *per se*.

The combination of LTG and NBV on acute or chronic administration elicited significant increase in the ST. It may be due to the action of the two drugs through different mechanisms or it could be due to drug interaction, which may be either pharmacodynamic or pharmacokinetic needing elucidation. It was thus observed that the NBV potentiated the anticonvulsant effect of LTG.

Cognition impairment is a problem frequently associated with the epilepsy.[18] An effort has been made to study the effect of LTG and NBV alone and in combination and it was found that there was no significant effect on cognitive impairment. The hippocampus has one of the denser inputs of adrenergic terminals (containing NE) in the CNS supporting the hypothesis that noradrenergic system plays a role in memory retrieval.[19]

Both, LTG and NBV, in the present study, when given individually or in combination, significantly impaired the motor activity as evident by reduction in grip strength in both acute and chronic dosages. This is in confirmation with earlier reports that documented invariable association of AEDs with motor impairment.[20]

Studies have reported that oxidative stress exacerbates epilepsy.[21] In the present study, NBV showed a dose-dependent inhibition of lipid peroxidation in the mice brain tissue. Our results demonstrate that NBV has antioxidant property, which is consistent with previous published reports.[22] Free radicals (OH or super oxide anion) are released inducing systolic and diastolic dysfunction, which could be prevented by NBV by, direct scavenging of ROS.[23]

Lamotrigine also demonstrated significant inhibition of lipid peroxidation in brain. Free radical generation can induce seizure activity by direct inactivation of glutamine synthase, thereby permitting an abnormal build up of excitatory neurotransmitter glutamic acid.[24] Further, there was also significant reduction of lipid peroxidation in the animals treated with the combination of LTG and NBV. It would be worthwhile to identify whether this inhibition of lipid peroxidation contributes to anticonvulsant action of these drugs.

Acute treatment with lower dose of LTG (1.5 mg/kg) and NBV (0.25 and 0.5 mg/kg) did not affect the GSH level; however, their combination exhibited augmentation in the latter. In contrast to acute effects, chronic administration of LTG and NBV alone or in combination resulted in elevation of the brain GSH levels.

Nebivolol showed antiepileptic effect in addition to its reported antihypertensive effect. Thus to conclude, the combination of LTG and the NBV may be effective in cases where associated risk factors, like hypertension, stroke, etc. might precipitate seizures. Since both the drugs are relatively safe in elderly, they can be used in elderly patients who are at the risk of seizures due to cerebrovascular disease. The results clearly demonstrate the additional benefits on epilepsy, memory, and muscle, brought about by the inclusion of NBV in the LTG regimen. However, more studies are required to find out the changes in both pharmacodynamic and pharmacokinetic parameters when the combination is used.
